# Potential Micronutrients and Phytochemicals against the Pathogenesis of Chronic Obstructive Pulmonary Disease and Lung Cancer

**DOI:** 10.3390/nu10070813

**Published:** 2018-06-25

**Authors:** Ting Zhai, Shizhen Li, Wei Hu, Duo Li, Shuguang Leng

**Affiliations:** 1 Department of Occupational and Environmental Health, School of Public Health, Qingdao University, Dengzhou Road 38, Qingdao 266021, China; zhaiting96@outlook.com (T.Z.); lszlishizhen@hotmail.com (S.L.); prettyvivian@126.com (W.H.); 2 Institute of Nutrition and Health, Qingdao University, Qingdao 266021, China; duoli@qdu.edu.cn

**Keywords:** respiratory disease, lung cancer, COPD, antioxidants, phytochemicals, vitamins, lung function, epigenetics, primary prevention, precision medicine

## Abstract

Lung cancer and chronic obstructive pulmonary disease have shared etiology, including key etiological changes (e.g., DNA damage and epigenetics change) and lung function impairment. Focusing on those shared targets may help in the prevention of both. Certain micronutrients (vitamins and minerals) and phytochemicals (carotenoids and phenols) have potent antioxidant or methyl-donating properties and thus have received considerable interest. We reviewed recent papers probing into the potential of nutrients with respect to lung function preservation and prevention of lung cancer risk, and suggest several hypothetical intervention patterns. Intakes of vitamins (i.e., A, C, D, E, B_12_), carotenoids, flavonoids, curcumins, resveratrol, magnesium, and omega-3 fatty acids all show protective effects against lung function loss, some mainly by improving average lung function and others through reducing decline rate. Dietary interventions early in life may help lung function reserve over the lifespan. Protective nutrient interventions among smokers are likely to mitigate the effects of cigarettes on lung health. We also discuss their underlying mechanisms and some possible causes for the inconsistent results in observational studies and supplementation trials. The role of the lung microbiome on lung health and its potential utility in identifying protective nutrients are discussed as well. More prospective cohorts and well-designed clinical trials are needed to promote the transition of individualized nutrient interventions into health policy.

## 1. Introduction

Lung cancer (LC) is the main cause of overall cancer mortality. Even with joint efforts for earlier detection and potent chemotherapy, the overall 5-year survival rate remains disappointing [1]. Chronic obstructive pulmonary disease (COPD) has become the fourth leading cause of death in the world [2], with identification as an independent LC risk factor [3]. Moreover, COPD and LC have shared etiology, such as aging, environmental and occupational exposure, inflammation and oxidation, and epigenetics changes [4,5]. The development of COPD and LC in ever smokers (i.e., current and former smokers) likely requires decades of repetitive exposure of the airway to cigarette smoke. Lung carcinogenesis consists of a cascade of key etiological changes prior to clinical cancer diagnosis, with some of them being identified as causal events in COPD genesis. Focusing on primary prevention by targeting these biological changes may thus be of high value for reducing the overall mortality in smokers. Moreover, chemoprevention strategies developed for one may help for the other, and interventions targeting on these communal pathogeneses may yield great success for the prevention of both.

Lung function has emerged as a promising target for prevention of both COPD and LC. Accelerated lung function decline with aging is both an indicator and cause of COPD, and studies have regarded lower or declining lung function (represented by forced expiratory volume in one second (FEV_1_)) as a strong indicator for all-cause and cardiovascular mortality [6,7]. Moreover, lung function has shown a strong correlation with LC risk in several meta-analyses [8,9]. A recent nested case-control study revealed the role of COPD severity indexes as independent predictors of LC [10]. Also, a more rapid FEV_1_ decline predicted subsequent LC incidence in smokers from the Pittsburgh Lung Screening Study [11]. Compared with other etiological changes, lung function is easier to measure in practice. Thus, intervention study targeting lung function decline may hold great premise of reducing the propensity for COPD and LC incidence.

Among all intervenable factors, smoking cessation has been shown to play a reliable role in preventing lung function decline and lung cancer incidence [12,13,14,15]. However, smoking cessation with long-term abstinence (over a year) is very difficult to achieve and the most comprehensive strategy combining behavior and pharmaceutical interventions for smoking cessation has little effect on the long-term abstinence rate [16]. In the meantime, the beneficial effect of nutrition on lung health has been well explored. Certain micronutrients and phytochemicals have proved to have anti-inflammatory and anti-oxidative properties, which can directly target the pathogenesis of lung function decline. Compared with smoking cessation, nutrition intervention can be relatively easily achieved and applied in the public. It thus has a promising future for the primary prevention of lung diseases.

In this review, we aimed to explore the protective effect of dietary micronutrients and phytochemicals on COPD and LC in the context of primary prevention, with special respect to their benefits for lung function development in early life and prevention of lung function decline and lung cancer risk in later life. Moreover, the role of lung microbiome on lung health and its potential utility in identifying protective dietary agents are discussed. Finally, perspectives on future studies regarding the development of precise nutrition interventions for high-risk populations are proposed.

## 2. Nutrients and Phytochemicals against Lung Function Attenuation during Adulthood and Old Age

Lung function decline is related to oxidative stress and inflammatory actions [5]. In later life, lung function is attenuated with aging, and the older population is more susceptible to environmental exposure [17]. Dietary micronutrients or phytochemicals with potent anti-oxidative or anti-inflammatory activities are thus promising agents with regard to the preservation of lung function, and the majority of current studies are focused on their protective effect in adulthood and old age. Fruit (e.g., apples, bananas), vegetables (e.g., tomatoes), herbal tea, fish, marine food, and white wine all showed lung function preservation potential in general or high-risk populations [18,19,20,21,22]. In contrast, cured meat and alcohol were associated with detrimental effects on lung function [23,24]. These effects were confirmed in large population-based dietary pattern studies [22,25,26,27]. Their protective properties were still significant even in combination with other foods and with risk of counteracting effects [28]. Studying diet patterns helps us to evaluate the effects of nutrition and identify high-risk populations. However, dietary patterns are difficult to change, and nutrient interventions seem more practical and can easily be achieved by taking supplements.

In 2013, Hanson et al. summarized observational literature about vitamins with respect to their role in lung impairment and COPD risk [29]. Besides the well-studied vitamins, evidence about fatty acids, minerals, and some antioxidant phytochemicals (non-provitamin A carotenoids and phenols) is emerging. In a recent cohort study, protective nutrients together with their hypothetical intervention patterns in lung function impairment were identified conjointly: intake of magnesium, folate, niacin, vitamins A and D, eicosapentaenoic acid (EPA), docosahexaenoic acid (DHA), and dietary fiber resulted in a FEV_1_-improving pattern; EPA and DHA intake slowed FEV_1_ decline [30]. Cross-sectional data found that FEV_1_ levels were positively associated with concentrations of serum antioxidant vitamins (vitamin A, vitamin C, vitamin E, β-cryptoxanthin) and minerals (selenium, normalized calcium, chloride, and iron) and inversely associated with potassium and sodium levels [31]. To better understand the different effects on lung function of various micronutrients or phytochemicals and the underlying mechanisms, further details are provided below.

### 2.1. Carotenoids and Vitamin A

Carotenoids are well-known for their antioxidative properties. Hanson et al. reviewed several cross-sectional studies and concluded a positive association between dietary or circulating provitamin A carotenoids and lung function. This association of serum carotene levels tended to be stronger among the community-dwelling and disabled population [29]. Though with no benefit in terms of LC risk, serum β-carotene and retinyl palmitate showed a positive association with FEV_1_ and forced vital capacity (FVC) in a supplementation trial [32]. In a longitudinal study among young adults, both higher baseline carotenoid levels and an increase in provitamin A carotenoid concentrations predicted a 15-year slower lung function decline [33]. Another cohort only found this association in serum β-carotene, suggesting a special role of β-carotene in lung health [34]. The inverse association between serum provitamin carotenoids and lung function decline was independent of dietary carotenoid intake [33]. In order to clarify their protective effect on lung function, it may be necessary to take both vitamin and provitamin A into account.

As for non-provitamin A carotenoids, the potential of lycopene on reducing FEV_1_ decline among ex-smokers has been indicated in a recent cohort study [19]. A systematic review in 2016 about lutein and respiratory outcomes reported a positive association in only three out of five included studies [35]. The authors further analyzed data from a prospective cohort and found the association was inverse in smokers [36]. Cross-sectional data also revealed an attenuated association between serum β-carotene and FEV_1_ among smokers [37]. A comparable degradation rate of carotenoids may help explain the conflicting results. Usually carotenoids are quickly oxidized after uptake and acquire oxidative properties. Most importantly, smoking can accelerate this autoxidation rate, thus enhancing the oxidative stress induced by smoking [38]. It is true that mostly smokers eat a suboptimal diet, and our information from observational studies cannot separate out this effect. However, taking into account the increased risk of LC among heavy smokers and asbestos-exposed individuals taking β-carotene supplementation in the Beta-Carotene and Retinol Efficacy Trial (CARET) and the Alpha-Tocopherol Beta-Carotene Cancer Prevention (ATBC) Study [39], it is possible that smokers may need to pay more attention to their dietary or supplemental intake of carotenoids.

Studies on vitamin A found rather conflicting results: In a supplementation trial, higher serum retinol levels predicted an increase in FVC among heavy smokers [32]. However, in cohort and cross-sectional studies, the relationships between lung function and both vitamin A intake and circulating levels were not consistent [18,19,40,41,42]. Retinols and carotenoids can both be regarded as vitamin A [43], and many studies did not distinguish the vitamin A they measured [18,40]. Retinol is a relatively unstable substance and retinol levels in lung tissue are not parallel to intake of vitamin A [44]. Thus, serum retinol levels may not be a good indicator for the study of dietary interventions. Future studies on vitamin A may need to clarify the source of vitamin A they apply.

In summary, carotenoids showed certain potential in the preservation of lung function among the general population. More clinical trials are warranted to prove their protective role against lung diseases. Moreover, smokers may need be cautious about their carotenoid intake. Evidence regarding to vitamin A is not consistent and future studies need to clarify the type and source of their indicators.

### 2.2. Vitamin C

Vitamin C, with its well-studied antioxidant activities, has been shown to protect lung tissue. Both dietary and serum levels of vitamin C were strongly associated with FEV_1_ and FVC in a 2010 meta-analysis [45]. Evidence has been reviewed among observational studies and positive associations were shown between vitamin C intake or concentrations and lung function among general population [29]. This significant, positive association of vitamin C was confirmed in longitudinal studies. There was a dose–effect relationship between vitamins C and E intake and lung function in a prospective cohort [46]. The smokers cohort also showed a positive association with FEV_1_ [30]. Moreover, a prospective study suggested a protective role of vitamin C on lung function decline [40]. This beneficial effect on FEV_1_ decline was strengthened in current smokers and quitters in another cohort [47]. Most recently, Garcia-Larsen et al. found a special role of vitamin C in slowing 10-year FVC decline [19]. With strong evidence suggesting its beneficial role in preserving lung function, vitamin C is likely to be of focus in future prospective cohorts and randomized control trials (RCTs) for lung health interventions. Focusing on the possible mechanism of vitamin C-mediated lung function preservation may yield great success in exploring potential targets for lung disease prevention.

### 2.3. Vitamin D

Recent studies have revealed many new sites of vitamin D and vitamin D receptors (VDRs) in organs including the immune system, suggesting its potential in defense in inflammatory reactions and infection. To date, there are limited studies investigating the association between dietary vitamin D and lung function. A review in 2013 included seven observational studies and only four showed an association with lung function [29]. A longitudinal study reported no association between vitamin D intake with both annual FEV_1_ and FVC decline [19]. Meanwhile, the intake of vitamin D was positively correlated to better FEV_1_ in another cohort study and population-based study [30,48]. Interestingly, a longitudinal study found an association between smokers with vitamin D deficiency (VDD) and steeper lung function decline [49]. Another study among older disabled community-dwelling women also found a positive association between serum 25(OH)D3 levels and lung function [50]. It is possible that vitamin D plays a special role on lung function in vulnerable populations. Another explanation for the conflicting results is that the skin is also a source of vitamin D [51]; vitamin D intake may not be an adequate indicator for total vitamin D exposure compared with serum 25(OH)D3. Due to the limited number of studies, the potential of vitamin D for lung function requires further research.

### 2.4. Vitamin E

Vitamin E exerts its potent antioxidant role mainly through its chain-breaking, membrane-repairing, and free radical-scavenging activity [52]. Dietary vitamin E intake is correlated with its levels in serum and lung tissues in humans [44,53]. Thus, research on dietary interventions with vitamin E and the effects on lung function decline is promising. A meta-analysis showed significant associations between vitamin E intake and circulating levels with lung function [45]. Later longitudinal studies also revealed a positive association with either FEV_1_ or FEV_1_ decline [31,54]. More interestingly, a cross-sectional study showed a dietary vitamin E intake decrease of 1 standard deviation had an impact on lung function decline that was equivalent to 1–2 years of aging. This revealed a promising area of study for dietary vitamin E interventions in mitigating aging-related lung function decline [55].

It is of note is that the meta-analysis did included several studies with non-significant results. There is also a supplementation trial that reported no effect of supplemental vitamin E on lung function [56]. Vitamin E has four typical isoforms: α-, β-, γ-, and δ-tocopherol. Recent studies had focused on various isoforms of vitamin E since there are studies reporting their opposing inflammatory mechanisms: an in vitro study suggested concentration of γ-tocopherol isoforms at 10% of that of α-tocopherol was able to ablate the anti-inflammatory effect of α-tocopherol [57]. Hanson et al. found that serum γ-tocopherol was inversely associated with FVC, while serum α-tocopherol showed no significant effect [54]. Since Grievink et al. also found no effect of α-tocopherol in their continuous efforts [58,59], it is possible that the conflicting results mentioned before are caused by a lack of differentiation of vitamin E isoforms. In addition, the antioxidative properties of vitamin E are partly mediated by and are synergistic with vitamin C [60]. Britton et al. reported a vitamin C-dependent association between vitamin E and lung function [61]. Thus, another explanation of the contradictory results is that many studies failed to take nutrient–nutrient interactions into consideration.

Collectively, vitamin E is a promising agent in lung function preservation, but more mechanistic studies are needed to clarify the most efficient isoform and offer support for further supplementation trials.

### 2.5. Minerals

Selenium, with its potent antioxidant properties, has been investigated in supplementation trials. Cassano et al. first reported the effect of selenium supplementation in an RCT but found no significance with respect to lung function, individually or in combination. However, the supplementation of selenium did show a beneficial effect on slowing the decline of FEF_25-75_ in current smokers [56]. Cross-sectional evidence revealed an independent, positive association with FEV_1_ in serum selenium, normalized calcium, chloride, and iron levels, while circulating potassium and sodium showed a negative association [31]. This association of serum selenium was strengthened in later cross-sectional studies among both smokers and the general population [37,62]. Intake of magnesium was found to be related to higher FEV_1_, but was not associated with FEV_1_ decline in longitudinal analyses [40]. This is consistent with findings from Leng et al. [28]. As for copper, a cross-sectional study suggests an association between increased blood copper levels and a decrease in FEV_1_ [62]. Due to limited literature numbers, the beneficial role of minerals in lung function is hard to affirm, and further well-designed, population-based prospective studies are needed.

### 2.6. Fatty Acids

Saturated fat has been reported to activate innate immune and interleukin-6 secretion in human body, thus negatively affecting the lung [63], while omega-3 polyunsaturated fatty acids (*n*-3 PUFAs) possess anti-inflammatory and anti-allergic properties, and may thus be beneficial to lung health. *N*-3 PUFAs EPA and DHA, which are present in fatty fish, marine oils, and other marine foods, are now well-studied in terms of their anti-allergic properties for primary prevention of allergic disease in offspring during gestation and in early life [64,65]. Several meta-analyses provided evidence that *n*-3 PUFAs might mitigate the detrimental effects of saturated fat by showing greater benefit of *n*-3 PUFAs in trials using a high-fat control formula [66,67]. However, those meta-analyses failed to show the benefits of *n*-3 PUFAs for lung function, as they are an instable indicator of pulmonary function. Due to the positive effects found, there is a trend of studying the potential of EPA and DHA for the prevention of lung function impairment among the general population. A nested case–control study firstly showed in men the inverse association between the proportion of fat in the diet and lung function (%FEV_1_ and %FVC), while total intake of fat, saturated fatty acids, monounsaturated fatty acids, *n*-3 PUFAs, and *n*-6 PUFAs showed no significance either in men or women [63]. However, cross-sectional evidence did show a positive association between intake of *n*-3 PUFAs and lung function [68]. The protective effect on lung function of EPAs and DHAs was clarified by Leng et al. as mainly working through reducing age-related FEV_1_ decline [30]. Moreover, higher DHA intake completely negated the detrimental effect on FEV_1_ decline by continuous current smoking. In summary, further evidence is needed to explore the impact of *n*-3 PUFAs and other fatty acids on lung health development. The different effect patterns on lung function between *n*-3 PUFAs and other protective nutrients may generate a new direction for the application of nutrientomics on lung function preservation.

### 2.7. Phytochemicals

Various dietary phytochemicals with potent antioxidative properties have been explored their potential as promising bioactives in addition to non-provitamin A carotenoids. Flavonoids are secondary metabolites from polyphenolic plants with significant antioxidative activities [69]. Longitudinal evidence showed that intake of flavonoid-rich herbal tea was associated with a slower 10-year decline in FVC [19]. Anthocyanin intake was found to have a strong protective effect against age-related lung function decline in a longitudinal study among elderly men [70]. Dietary intake of total flavonoids, catechins, and pro-anthocyanidins was shown to have an inverse relationship with lung function decline in several cross-sectional studies [68,71].

Curcumins, phenols naturally found in turmeric, possess potent anti-oxidant and anti-inflammatory actions. Regarding to their potential for lung health, Ng et al. reported a significant positive association together with a dose–response relationship between curcumin-rich curry intake and FEV_1_, FEV_1_/FVC in elderly Chinese adults [72]. The pulmonary function in smokers with curcumin-rich curry intake was almost equal to that in non-smokers, suggesting a special benefit of curcumins for smokers.

Recently, alcohol intake has been proven to have a U-shaped relationship with COPD mortality [24,73]. Studies have suggested that alcohol consumption contributes to impaired lung function as an oxidative stressor [74]. Besides the alcohol dose consumed, the phenolic compounds found in wine were shown as another possible determinant for this U-shape effect, due to their potential as radical scavengers [75,76]. Wine drinking has been shown possibly associated with better lung function [20,77], and resveratrol as a typical phenol in wine is being frequently linked to reduced COPD risk [78]. Siedlinski et al. found a positive association between intake of resveratrol and FVC but failed to establish causation in longitudinal aspects [20]. To further explore benefits of wine, more studies should be conducted regarding the role of resveratrol and other possible phenolic compounds. The public should be informed about the possibly harmful effects of alcohol use on lung health.

Due to various categories and potent anti-oxidative properties of phytochemicals, future studies are warranted to clarify the potentially different outcomes and underlying mechanisms of them.

**Figure 1:**
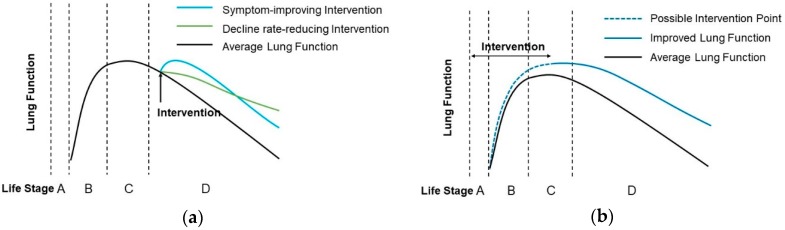
Hypothetic model for the effect of dietary interventions on lung function over the lifespan. (**a**) Symptom-improving intervention and decline rate-reducing intervention patterns by dietary nutrients or phytochemicals in the older life stage; (**b**) A conceptual model about nutrient intervention by life stage before lung function peak for lung function preservation over the lifespan. Life stage A: the maternal and prenatal period; B: childhood; C: young adulthood; D: older life.

Leng et al. reported two hypothetical patterns regarding role of nutrient intervention for lung function improvement in later life: one is symptom-improving pattern, and another is rate-reducing pattern [30]. We summarized the longitudinal evidence above and introduced carotenoids into the symptom-improving pattern, while lycopene, flavonoids, vitamin C and E formed part of the rate-reducing pattern (Figure 1a).

## 3. Dietary Intervention in Early Life

Recently, failure to achieve optimal peak function caused by adverse exposure in early life has been proved as a cause of chronic respiratory disease, in addition to an accelerated decline in lung function with aging in adulthood [17,79]. Numerous studies have shown that lower lung function in early life stage strongly predicts permanent lung function decline in later life [17,80,81,82]. Several risk factors like maternal smoking and childhood respiratory infections have been identified [80]. Interventions to improve lung function from the prenatal period to young adulthood before peak lung function is reached may thus be a key component in prevention of COPD and LC, with the potential to maximize respiratory reserve in later life [17]. Similar to that of older life stage, rate of smoking cessation is not satisfactory among pregnant women [83,84,85]. Due to the well-studied role of nutrition on lung function in adults, there is a growing interest in exploring its beneficial effects on lung health in early life.

Consistent with findings in adults, healthy diets (including higher consumption of fruit, vegetables, and grains, lower dairy and sweet intake) were associated with better lung function in childhood and young adulthood [86,87]. A significant association between higher lutein/zeaxanthin levels in young adults and lower FEV_1_ and FVC decline in later life was found, which further strengthens the theory that early life interventions with certain micronutrients or phytochemicals might yield great success in the prevention of disease later in life [33]. Another cross-sectional study among Chilean young adults found higher total fruit and catechins intake was associated with better FVC [68]. Gilliland et al. found deficits in lung function were associated with lower magnesium intake among children, while a similar association with lower potassium intake was also found in girls [88].

Besides findings in childhood and young adulthood, the effect of nutrition during pregnancy and lactation period on lung development of offspring is another focus. Vitamin A supplementation before and during pregnancy and in the immediate postpartum period was reported to be associated with better lung function among pre-school aged children in a population with chronic vitamin A deficiency [89]. Newborns of women smokers with vitamin C supplementation during pregnancy showed improved newborn pulmonary function. Also, the higher trend of respiratory compliance after one-year follow-up suggests the persistent beneficial effect of vitamin C supplementation in the offspring of smokers [90]. Later in a supplementation trial, significant restoration was found of both genomic and tissue-specific methylation related to maternal smoking. More importantly, the restoration was associated with lung function in newborns [91]. Colostrum omega-3 PUFAs levels were found to be associated with mean FEV_1_ and FEV_1_ /FVC ratio at 12 years in a high-risk birth cohort [92]. In the meantime, maternal sugar intake was not found to have an association with childhood lung function in another cohort [93].

Based on these new evidences regarding beneficial nutrients with lung function development in different stages of early life, we may add a third nutrient intervention pattern of lung function development into our hypothetical patterns: an intervention encompassing maternal, prenatal, and early life stages might be beneficial to reserve of lung function over the lifespan (Figure 1b). Studying those patterns might generate new thoughts and directions with regard tonutrientomic studies, and large-scale, long-term follow-up cohorts are warranted.

## 4. Nutrients Intervention in Lung Cancer Chemoprevention

Lung carcinogenesis is a multistage process that is driven by the acquisition of a constellation of genetic and epigenetic changes resulting in the activation of oncogenes, the silencing of tumor suppressor genes, and ultimately the clonal expansion of malignant cells. Previous experience has suggested that targeting the pathogenic process which is either reversible (e.g., epigenetics) or stoppable before the cellular malignancy is established is ideal for the chemoprevention of lung disease. In this section, we summarized recent studies focusing on role of nutrition for two actionable targets including epigenetic changes and DNA damage repair in LC and COPD development.

### 4.1. Nutrients Targeting Epigenetics

Epigenetics, including DNA methylation, non-coding RNA expression, chromatin modeling, and post transcriptional regulators are key in lung carcinogenesis. Methylated promoter detection in sputum was regarded as a reliable biomarker for early stage LC diagnosis and poorer prognosis [94,95]. Conversely, genome-wide hypomethylation increases with cancer progression [96]. Most recently, gene methylation in sputum also showed a strong association with lung function decline in the Lovelace Smokers Cohort [11]. Several nutrients (e.g., B vitamins, vitamin C) [97,98] and phytochemicals [99,100] have shown effects in modulating epigenetics, and dietary intervention is thus promising for chemoprevention of COPD and LC.

Folate is a typical methyl donor involved in one carbon metabolism. Insufficiency of folate can result in promoter hypermethylation of tumor suppressor genes, as suggested by a review in 2012 [101]. Folate intake has showed a beneficial effect on lung function preservation in a smoker cohort [30]. Stidley et al. had identified folate, leafy green vegetables, and multi-vitamin use as three protective factors against acquisition of gene methylation in lungs of non-Hispanic whites [102]. Later in the same cohort, another five factors including vitamin D, B_12_, manganese, magnesium, and niacin were also identified as protective nutrients against gene methylation [103]. A nested case-control study found that serum folate was associated with increased methylation levels of ras association domain family 1 isoform A (RASSF1A) and methylene tetrahydrofolate reductase (MTHFR), while methionine showed an inverse association with that of RASSF1A [104]. As for genomic DNA methylation, a study found higher ascorbic acid accumulation in human small cell carcinoma (SCC) tissues, in association with global methylation of DNA [105]. Later, the authors found lower folate and vitamin B_12_ concentrations in SCC tissues, which were also related to global DNA methylation [106]. Taken together, results from SCC tissues suggests that folate and vitamin B_12_ availability is a limiting factor for global DNA methylation, while vitamin C in SCC tissues may help facilitate the global methylation rate.

Histone modulation (i.e., acetylation of histones H3 and H4) leads to chromatin modeling, which alters gene transcription (including for several anti-oncogenes and DNA repair genes) [107]. Histone deacetylases (HDACs) are important for gene transcription [108]. Histone modulations have been identified as an important chemoprevention target and HDAC inhibitors have been regarded as promising anti-cancer agents [109]. Diet is a modifiable factor for histone modulations and a high-fat diet is associated with chromatin modifications in animal models [110,111]. For now, short-chain fatty acids (SCFAs) are well-studied HDAC inhibitors, and dietary SCFAs were shown to be associated with the inhibition of HDAC activity in vitro [112]. Studies with respect to LC were limited to the cellular level, but showed great potential in cancer prevention by inhibition of HDAC activity [109,113]. Besides SCFAs, dietary *n*-3 PUFAs also showed EZH2-inhibiting properties in cancer cells. Nutrient interventions related to HDAC activity are promising, but require further proof using population-based studies.

### 4.2. Nutrients Targeting DNA Damage Repair

Cigarettes contain a huge number of free radicals, and polycyclic aromatic hydrocarbons (PAHs) play a major role in lung carcinogenesis. Free radicals cause cell and DNA damage and thus elevated levels of oxidative stress. Durham et al. have reviewed pathways through which oxidative stress initiates carcinogenesis: DNA damage (point mutations, single-stand breaks (SSBs) and double-strand breaks (DSBs)), and DNA cross-linking [5]. Since carcinogenesis cannot be explained only by genetic alterations and genetic mutation is not stoppable, nor reversible, targeting of DNA repair or reducing DNA damage is more practical for chemoprevention. Studies on the effect of antioxidant nutrients on LC have made some progress with regard to mitigating oxidative DNA damage.

One of the most studied and potent PAH is B(a)P. Significantly reduced B(a)P-DNA adducts were reported in women receiving vitamin C and α-tocopherol supplementation [114]. Subsequent experimental studies also revealed positive results in smoke-induced DNA damage. Liu et al. using β-cryptoxanthin supplementation showed decreased 8-hydroxy-20-deoxyguanosine (8-OHdG) in the lungs of animal models [115]; and combined antioxidant supplementation (β-carotene, α-tocopherol and ascorbic acid) also protected single-strand breaks in ferret models [116]. Further population-based studies are warranted to clarify the beneficial effects of antioxidant nutrients on oxidative DNA damage and the following risk of lung disease.

### 4.3. Lessons from Supplementation Trials

Population based studies have shown reduced LC risk with a healthier dietary intake of fruit, vegetable, nuts, soy, and micronutrients including vitamin B6, folate, β-cryptoxanthin, and vitamin D [117,118,119,120,121,122,123,124,125]. Interestingly, in contrast to these inverse associations, large vitamin supplementation trials showed no protective effect of those cancer-preventive nutrients, or even poorer outcomes (e.g., increased LC risk and all-cause mortality) [39]. Higher doses, shorter durations, nutrient intake of other nutrients, formulation variations, and the timing of interventions were implied as confounding factors in these trials [126]. Indeed, lots of supplementation trials selected doses beyond the recommended daily allowance (RDA) or even the upper tolerable intake level (UL) each day. Besides, daily intake of certain nutrients has been elevated by food supply in the general population due to health policy [127]. High-dose supplementation is thus likely to show limited or even harmful effects when exceeding the RDA and UL. More importantly, since the protective effect of certain nutrients on LC risk was mainly observed in combination with other nutrients, it is possible that single nutrients cannot work in the real world. Humans never eat just one single nutrient, and biological processes require the constellation of multiple nutrients. Moreover, as numerous studies have validated the protective role of nutrients in lung function decline, which is an early event in LC, the negative results of those supplementation trials are likely due to the quite short follow-up duration. After all, supplementary nutrients showed protective effects on molecular mechanisms and lung function in those LC chemoprevention trials [32]. Evaluation of those validated early events, such as DNA methylation in sputum and FEV_1_, may thus be of high value for efficacy assessment of those nutrient supplementation trials.

In light of lessons from previous RCTs, multiple-nutrient supplementation trials, which simulate the complex intake in the real world with a proper duration and dose, are much more promising to clarify roles of nutrients in lung carcinogenesis. RCTs themselves are needed to generate scientific evidence to support manufacturing and health policy.

## 5. Nutrients and Microbiomes

The lung was thought to be a sterile organ until the first report identifying a lung microbiome in healthy subjects [128]. Since then, numerous studies have explored the diverse microbiota in the human lung by using molecular techniques and have found evidence that lung microbiomes might change in COPD pathogenesis [129]. COPD is characterized by small airway inflammation, which intensifies with pathogenesis. Gammaproteobacteria, a typical lung microbiome class which are represented by *Pseudomonas aeruginosa*, were found grow rapidly under chronic inflammatory conditions and increase in lungs during disease [130,131]. Studies probing into the relationships between the lung microbiome and inflammatory response found there might be a feedback loop: gammaproteobacteria feed on inflammatory products while encoding components to promote inflammation [132]. Several studies have demonstrated accelerated lung function decline with recurrent lower respiratory tract infections, together with the exacerbation of lung function decline by bacteria [133,134]. Lung microbiomes may thus be of great importance in the pathogenesis of lung function impairment and COPD. Nutrition can be promising for the prevention of lung function decline caused by possible imbalance of the lung microbiome. Since several nutrients possess anti-inflammatory effects, supplementation of them might be helpful for improving lung function by breaking the feedback loop between the abnormal lung microbiome and inflammatory response. Lung microbiome composition was found change with dietary vitamin D, and serum 25(OH)D levels were inversely associated with *Pseudomonas* in the lung of animal models [135]. However, for now, limited studies have investigated the benefit effect of nutrients on lung microbiome balance, due to the lack of more accurate knowledge about the lung microbiome and its role in the pathogenesis of lung disease.

## 6. Perspectives

**Figure 2:**
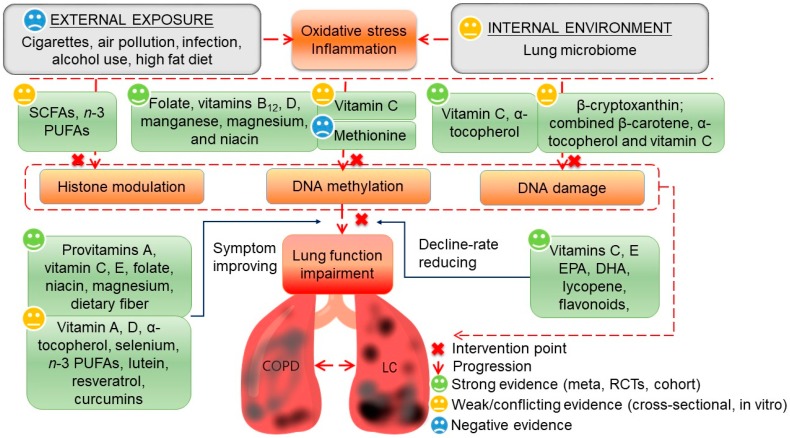
Summary of potential protective micronutrients and phytochemicals in the pathogenesis of chronic obstructive pulmonary disease (COPD) and lung cancer (LC). External and internal factors lead to oxidative stress and inflammation and thus initiate COPD and LC pathogenesis. Interventions against external exposure are not satisfying, while targeting the lung microbiome is promising. Recent studies have revealed strong evidence on protective nutrients in DNA methylation and damage but studies on histone modulation are limited to animal or cell experiments. Epidemiological studies about micronutrients and phytochemicals in later key event lung function impairment are abundant, and have identified different intervention patterns, including symptom improvement and a decline rate-reducing pattern. PUFA: polyunsaturated fatty acid; EPA: eicosapentaenoic acid; DHA: docosahexaenoic acid; SCFA: short-chain fatty acid; RCT: randomized controlled trials.

We have reviewed evidence about potential protective micronutrients and phytochemicals targeting key pathogenic events in the development of COPD and LC (Figure 2). Adding those protective dietary agents into the daily diet with proper doses may be of significance in improving the average lung function among the general population and thus primary prevention of COPD and LC.

A diverse range of external and internal factors can lead to impaired lung growth [17,136]. Unfortunately, many risk factors ,including cigarettes and air pollution, cannot be avoided voluntarily or successfully [16,137]. Precision prevention targeting pathogeneses of COPD and LC is thus important for these affected individuals. In earlier sections we have reviewed mechanistic and epidemiological evidence about micronutrients or phytochemicals in the prevention or mitigation of detrimental effects caused by smoking or air pollution or childhood disadvantage. This evidence is of high value for identifying precision targets for nutrition intervention among high risk populations. Moreover, nutrigenetics and nutrigenomics are now promising methods that should be taken into consideration in designing nutrition intervention practice [138]. Further investigations about interactions between genetic variations and dietary effects may thus offer a better understanding of potential targets for precision prevention with nutrients. Our summary of potential protective nutrients and their effects on LC carcinogenesis at the epigenetic level may also offer some new thoughts for future nutrigenomics research. For now, quite a few supplementation trials yielded negative results, signaling the need for personalized precision approaches with proper targets and doses. This type of research could open a new door for COPD and LC interventions in the era of precision medicine.

It should be noted that circulating levels of nutrients or phytochemicals may not represent their intake. In our review, both intake and circulating levels were discussed and summarized, with some inconsistent results. For instance, serum carotenoids showed strong association with lung function preservation, while results about their intake were rather conflicting. The review from Hanson et al. discussed the correlation between intake and serum level of vitamins with regard to their possible effects in the lung [29]. They suggested that serum vitamin C and E was not reflective of intake, but we did find both intake and circulating levels were associated with lung function. In cohort and cross-sectional studies, intake levels of certain nutrients are mostly obtained from food questionnaires, which may not be as precise as measured circulating levels. Nevertheless, circulating levels are also affected by many factors and also may not offer direct evidence in terms of daily intervention dose. To further explore their benefits, the advantages and disadvantages of both indicators may need to be taken into account in future study designs.

Supplementation trials can generate direct evidence about benefits of certain micronutrients or phytochemicals. As for cohort studies, even if they can build a temporal relationship, causation cannot be inferred. However, for now, most studies are limited to large observational or cohort studies and conducting those studies does help us identify possible protective nutrients or phytochemicals and offer evidence for further confirmation in clinical trials. Besides, mechanistic studies are also key in providing experimental evidence to determine causation. In this review, we reviewed evidence about micronutrients and phytochemicals targeting early molecular pathogeneses or later lung function impairment, thus offering new perspectives for the future design of clinical trials.
